# Metamaterial-Inspired Electrically Compact Triangular Antennas Loaded with CSRR and 3 × 3 Cross-Slots for 5G Indoor Distributed Antenna Systems

**DOI:** 10.3390/mi13020198

**Published:** 2022-01-27

**Authors:** Arshad Karimbu Vallappil, Bilal A. Khawaja, Mohamad Kamal A. Rahim, Muhammad Naeem Iqbal, Hassan T. Chattha

**Affiliations:** 1 Advance RF and Microwave Research Group (ARFMRG), School of Electrical Engineering, Faculty of Engineering, Universiti Teknologi Malaysia, UTM Johor Bahru, Johor Bahru 81310, Johor, Malaysia; mdkamal@utm.my (M.K.A.R.); naeem.iqbal@graduate.utm.my (M.N.I.); 2 Department of Electrical Engineering, Faculty of Engineering, Islamic University of Madinah, P.O. Box 170, Madinah 41411, Saudi Arabia; 3 Department of Electrical Engineering, PN-Engineering College (PNEC), National University of Sciences and Technology (NUST), Karachi 75104, Pakistan; 4 Department of Information Technology, Focus College, Kelowna, BC V1Y 8A6, Canada; chattha43@hotmail.com

**Keywords:** metamaterial (MTM), fifth-generation (5G), indoor distributed antenna systems (IDAS), complementary split-ring resonator (CSRR), unit-cells, cross-slot MTM

## Abstract

In this article, two distinct kinds of metamaterial (MTM) antennas are proposed for fifth-generation (5G) indoor distributed antenna systems (IDAS). Both antennas operate in the sub-6 GHz 5G band, i.e., 3.5 GHz. The antenna’s radiating structure is based on a combination of triangular and rectangular patches, as well as two complementary split-ring resonators (CSRR) unit-cells etched on the top layer. The bottom layer of the first MTM antenna is a complete ground plane, while the bottom layer of the second MTM antenna is etched by a 3 × 3 cross-slot MTM structure on the ground plane. The use of these structures on the ground plane improves the antenna bandwidth. The proposed antennas are designed using two different substrates i.e., a high-end Rogers thermoset microwave materials (TMM4) substrate (*h* = 1.524 mm/*ε_r_* = 4.5/*tan δ* = 0.002) and a low-end flame-resistant (FR4) epoxy glass substrate (*h* = 1.6 mm/*ε_r_* = 4.3/*tan δ* = 0.025), respectively. The antenna designs are simulated using CST microwave studio, and in the end, the antenna fabrication is performed using FR4 substrate, and the results are compared. Furthermore, parametric analysis and comparative studies are carried out to investigate the performance of the designed antennas. The simulated and measured results are presented for various parameters such as return-loss, gain, and radiation pattern. The two MTM antennas have an overall dimension of 18 × 34 mm^2^, demonstrating that the proposed design is 60 percent smaller than a standard microstrip patch antenna (MPA). The two proposed MTM antenna designs with complete ground plane and 3 × 3 cross-slot MTM on the bottom layer using FR4 substrate have a measured gain/bandwidth characteristic of 100 MHz/2.6 dBi and 700 MHz/2.3 dBi, respectively.

## 1. Introduction

The increasing number of individuals using mobile phones, tablets, and other handheld and wearable wireless devices has accelerated the development of wireless communication technologies and systems, including antennas. In particular, for next-generation communications, which require a lightweight antenna with high-gain to improve system performance, the compact antenna must have a high-gain and a large bandwidth to meet the increased demand for excellent communication quality with millions of users.

The demand for high-speed and broadband data services among mobile users is skyrocketing, and more than 80% of mobile data traffic is handled indoors, such as in commercial buildings and airports [1]. As a result, indoor distributed antenna systems (IDAS) are becoming increasingly vital for providing wireless communication coverage in high-traffic and dense indoor environments [2]. Because the majority of IDAS antennas are installed on the ceilings or walls of indoor spaces, they must have a low-profile, large bandwidth, and high-gain [3].

The printed microstrip patch antenna (MPA) is a suitable choice because of its low-profile, but it has the drawback of having a narrow bandwidth [3]. Different types of broadband printed antennas have been proposed to improve bandwidth for broadband applications, including modified fractal antennas [4,5], planar elliptical antennas [6,7,8], notched trapezoidal monopole antennas [9], dual band-notched circular ring antennas [10], a half-disc and a half-ellipse antenna [11], and a back-to-back triangular-shaped patch antenna [12]. However, all the previously proposed antennas suffer from low-gain at low frequency. As a result, low-profile broadband antennas with good gain that can cover the whole sub-6 GHz 5G frequency band are in high demand [1]. A metamaterial (MTM) antenna design technique, on the other hand, can be utilized to overcome the limits of traditional MPA antenna design schemes and is often focused on enhancing antenna performance, bandwidth, weight, directivity, efficiency, and other parameters [13,14,15,16].

In recent years, the scientific community has shown an increased interest in the study of MTMs [13,14,15,16]. The MTMs are the artificial, effectively homogeneous electromagnetic structures that exhibit novel electromagnetic properties that are not typically found in nature [17,18,19]. A structure whose average cell size *p* is much smaller than the quarter wavelength (*p* < *λ_g_*/4) is known as an effectively homogeneous structure. MTMs are a type of artificially constructed material dependent on periodical structures. These materials have negative permittivity (*ε*) and permeability (*μ*) at the same time, thereby known as double negative metamaterials (DNMs) [20,21,22]. In 1999, a scientist named Pendry et al. [23,24] first introduced negative-*ε*/positive-*μ* and positive-*ε*/negative-*μ* structures. Pendry introduced periodically located metallic thin-wires (TW) to achieve negative permittivity. Later, his team proposed spilt-ring resonators (SRRs) to get negative permeability [23,24]. In [25], Smith et al. combined Pendry’s TW and SRR structures into the composite structure. The TW and SRR structure develops negative-*ε* and negative-*μ*, respectively. This leads to generating left-handed-MTM (LH-MTM).

Some researchers focused their studies on the multi-band MTMs. The first kind of multi-band MTMs is based on well-designed metallic patterns, such as the ‘S-shaped’ [26], the ‘H-shaped’ [27], and the ‘Z-shaped’ [28] MTMs. An advanced, well-designed metallic pattern mode is fractal shapes, such as the nested ‘U-shaped’, and the ‘tree-shaped’ [29] MTMs. Another kind of multi-band MTMs is implemented with a combination of different single-band MTM units. In [30], similar patterns are printed on different substrates, while in [31], SRRs with different opening directions are printed on 3-layers substrates.

A few other researchers focused on the studies on tunable MTMs. There are multiple methods to implement tunable characteristics, such as loading varactors [32], applying ferrite-slabs [33] or liquid-crystals [34], and mechanically changing the structures [35]. Among these methods, varactor-loaded tunability is the most applicable solution. Some researchers focused on the studies to improve the gain of antenna by using MTM structures. The MTM structure has been used as a superstrate or loading MTM around the radiating patch. Other researchers used the MTM as a defective ground structure to improve the overall bandwidth of the antenna [36]. MTM structure properties enable a lot of applications in microwave and optical frequencies, including absorbers [37], energy harvesting [38], meta-lenses [39], and cloaking [40].

Therefore, this paper presents two MTM antenna designs using a complementary SRR (CSRR) and Cross-slot structure. The radiating structure of the antenna is designed based on the combination of triangular and rectangular patches and two CSRR unit-cells etched on the top layer. For the first antenna, the bottom layer is a complete ground plane, i.e., perfect electric conductor, and for the second antenna, the bottom layer is etched by 3 × 3 cross-slots MTM on the ground plane. The two antennas have been designed using two different substrates, i.e., a low-end flame-resistant (FR4) glass epoxy substrate and a high-end Rogers TMM4 substrate. Both antenna designs are compared in terms of performance, such as gain, bandwidth, radiation pattern, and S-parameters. The proposed antennas are operating at 3.5 GHz. Many enticing features of the proposed antenna include large bandwidth, high gain, and directional radiation pattern, making them an ideal candidate for 5G indoor distributed antenna systems (IDAS). The rest of this article is carried out as follows: The MTM unit-cell design, proposed antenna design configuration, and its simulation results, including parametric analysis, are briefly discussed in Section 2. Section 3 concisely discussed the analysis of the proposed design with the different substrates. The two MTM antenna simulated and measured results, including return-loss (*S*_11_), E- & H-plane radiation patterns, and gain characteristics, are discussed and summarized in Section 4. Section 5 compares the results of the two proposed MTM antennas with existing research articles and provides its findings, while Section 6 draws conclusions.

## 2. Proposed Antenna Design

**Figure 1:**
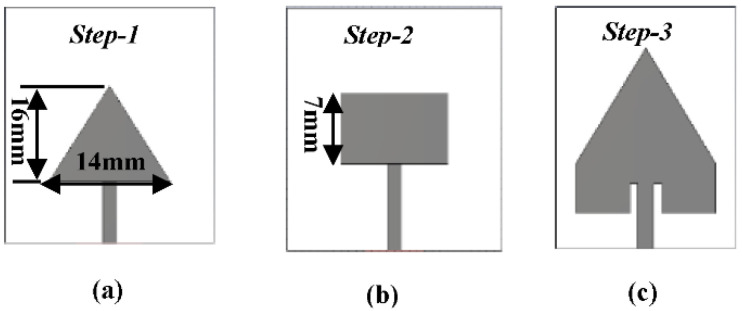
Step -by-Step design of proposed antenna (**a**) Triangular Patch (**b**) Square Patch (**c**) Combined Triangular and Square patch.

The proposed antenna design starts with a triangular patch of side-length 14 mm, as shown in Figure 1a. The resonant frequency can be calculated based on Equation (1) without the fringing effect [41]. The parameters *c*, *a*, *ε_r_* represents the velocity of light, triangular patch side-length, and dielectric constant, respectively.

(1) $$f_{r}=\frac{2c}{3a\sqrt{\varepsilon_{r}}}$$

**Figure 2:**
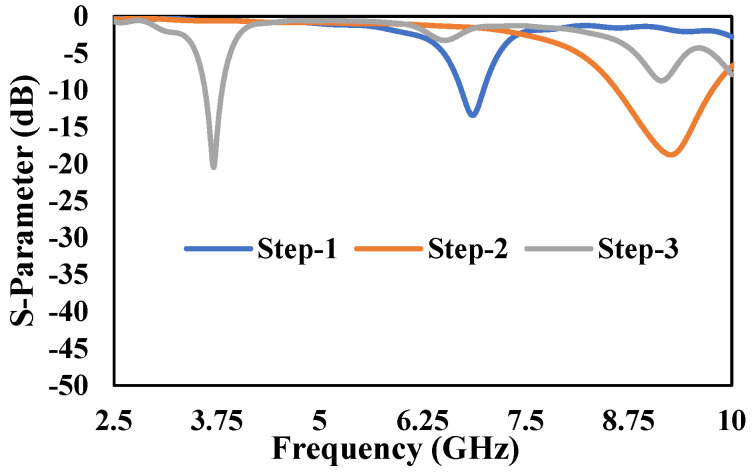
Step-by-Step design S-parameter response of Proposed antenna.

The resonant frequency may be determined with better accuracy if *a* and *ε_r_* in the above equation are replaced by effective dielectric constant *ε_eff_* and *a_eff_*, which are given by [42]. Finally, as per the calculation, the triangular patch antenna (TPA) resonates at 7 GHz, and the results are verified by simulating the TPA using CST microwave studio based on FR4 substrate having an *ε_r_* and thickness (*h*) of 4.3 and 1.6 mm, respectively, as shown in Figure 2.

(2) εreff=(εr+1)2+(εr−1)2(1+12×hW)−12=3.72

(3) $$a_{eff}=a+\frac{h}{\sqrt{\varepsilon_{r}}}=14.77$$

(4) $$f_{r}=\frac{2c_{0}}{3a_{eff}\sqrt{\varepsilon_{reff}}}=7\;\mathrm{GHz}$$

The next step is to design a rectangular patch antenna (RPA) having the same width (*W*) of TPA and length (*L*) of 14 mm and 7 mm, respectively, as shown in Figure 1b. In order to get the same width of TPA, we selected 9 GHz as the resonating frequency for the RPA, and the simulated results are illustrated in Figure 2. In the third step, we combined TPA and RPA, as shown in Figure 1c. The 3.5 GHz frequency band has been chosen in this study for 5G technology [43]. Moreover, the 700 MHz, 3.5 GHz, and 26/28 GHz have also been identified as potential frequency bands for the initial deployment of 5G technology in Malaysia [44]. The mathematical resonant frequency of TPA (*f*_1_) and RPA (*f*_2_) is 7 GHz and 9 GHz, respectively. The new resonant frequency after the combination of TPA and RPA will be (*f*_1_ + *f*_2_)/4, i.e., is equal to 4 GHz. After an in-depth parametric simulation study using CST microwave studio, it can be seen that the combined structure resonates at 3.75 GHz, as shown in Figure 2. In order for the comparison, the size of the individual RPA to resonate at 3.75 GHz will be 24.5 × 19.5 mm^2^. So, the total area of individual RPA will be 478 mm^2^. The proposed antenna patch has a total area of 195 mm^2^, which indicates a size reduction of 60% compared to the individual RPA.

### 2.1. CSRR MTM Structure Inserted to Proposed Antenna

**Figure 3:**
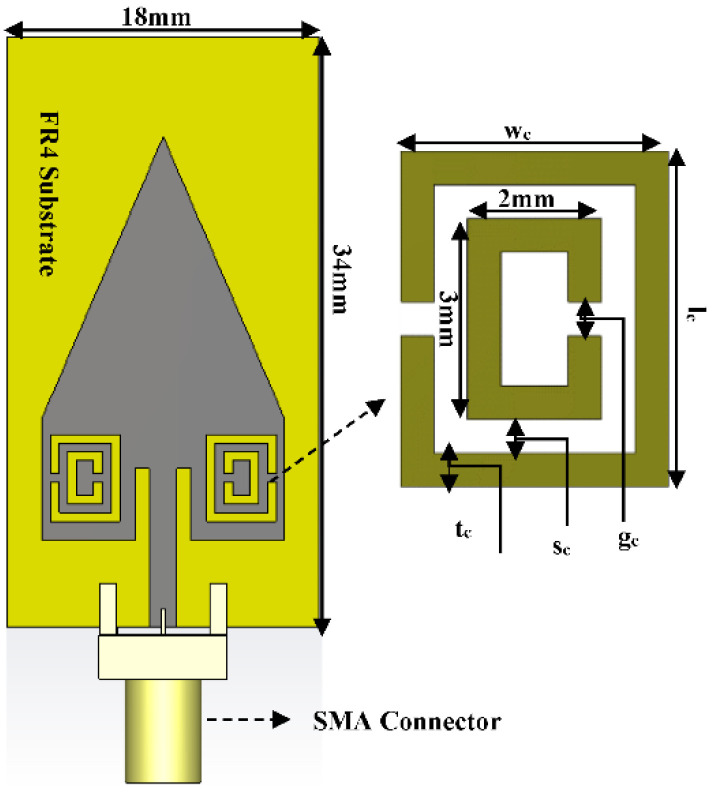
Layout of proposed antenna design.

**Figure 4:**
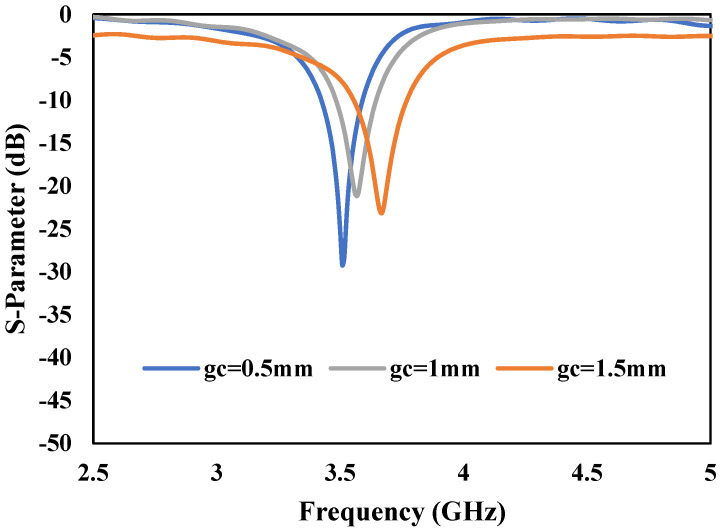
S-Parameter response of proposed antenna by varying the dimensions (*g_c_*) of CSRR unit-cell which has fixed value of 0.5 mm for *t_c_* and *s_c_*.

A CSRR MTM structure has been inserted into the proposed antenna’s radiating patch [45]. The structure and dimension of the CSRR structure used in this study is shown in Figure 3. The overall dimension of the proposed antenna after inserting the CSRR structure is also shown in Figure 3. The insertion of the CSRR structure shifts the center frequency of the proposed antenna to 3.5 GHz, as shown in Figure 4. The return-loss (*S*_11_) graph is plotted by varying the dimension of the CSRR parameter ‘*g_c_*’ to 0.5 mm, 1 mm, and 1.5 mm, respectively, as shown in Figure 4. From the graph, it can be observed that the frequency is shifted to the left by reducing the gap ‘*g_c_*’ and shifted to the right by increasing the gap. Finally, the desired results were achieved for the CSRR parameters at *g_c_* = 0.5 mm, *t_c_* = 0.5 mm, and *s_c_* = 0.5 mm. The results are highlighted with a solid blue-line in Figure 4, and it can be observed that the proposed antenna shows a bandwidth of 150 MHz and a return-loss of −30 dB at 3.5 GHz. The optimized dimensions of the CSRR unit-cell are shown in Table 1.

### 2.2. Metallic-Cross Metamaterial Structure 

**Figure 5:**
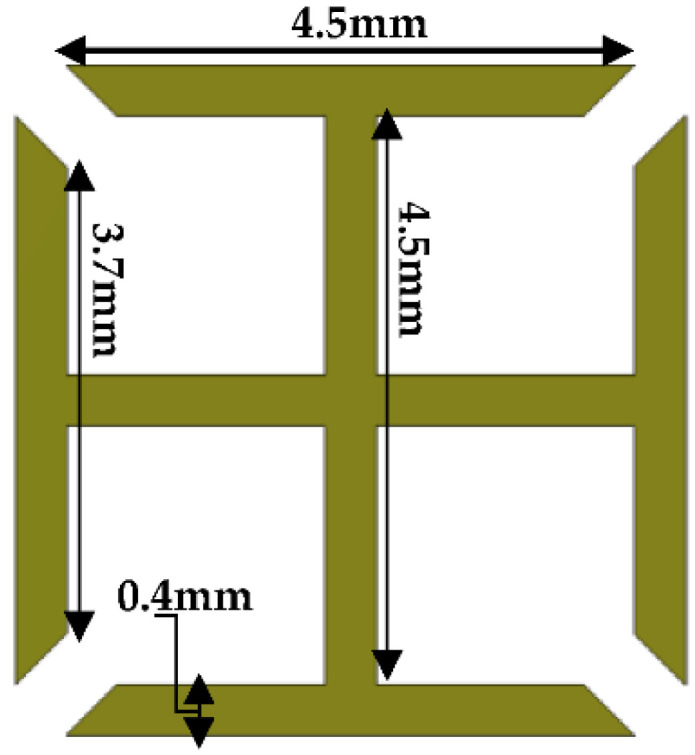
Metallic-Cross MTM unit-cell structure.

**Figure 6:**
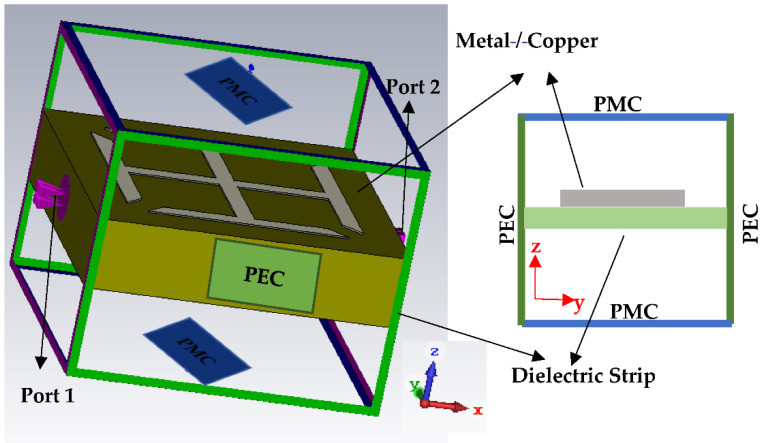
Boundary conditions and excitations applied to a single unit-cell.

The proposed metallic-cross MTM unit-cell is initially simulated by putting it in a rectangular waveguide to investigate the MTM properties. The structure of the proposed metallic-crosst MTM unit-cell is shown in Figure 5. The proposed MTM unit-cell used to load in the ground plane of the antenna, is expected to demonstrate the LH-MTM properties. For this purpose, a metallic-cross unit-cell printed on FR4 substrate has been designed and simulated as MTM with the help of CST Microwave studio. In this case, perfect boundary conditions have been applied to a unit-cell comprising only one metallic-cross, having two sides of the unit-cell (along the y-axis) with a perfect electric conductor (PEC) as a boundary condition, and the other two (along the z-axis) with a perfect magnetic conductor (PMC), as can be seen in Figure 6. The remaining two sides of the unit-cell (along the x-axis) are used as wave ports for excitation and radiation purposes. Further details on boundary conditions and excitations assignment to a unit-cell are shown in Figure 6. 

**Figure 7:**
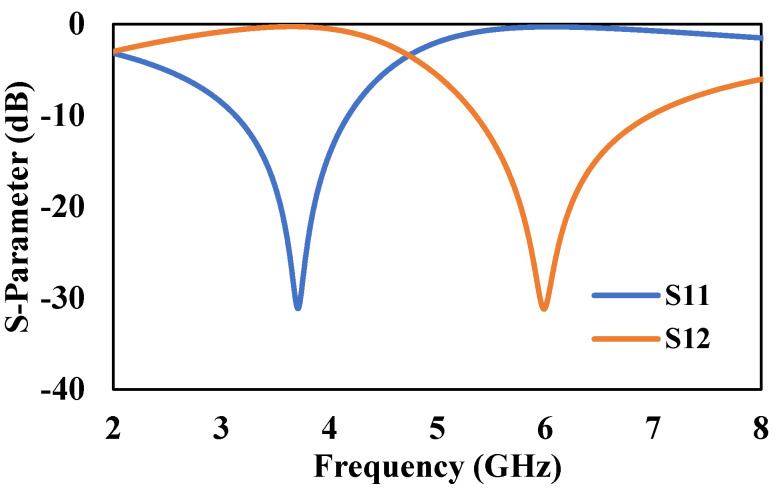
S-Parameter response of metallic-cross Unit-cell.

The S-Parameter response of the unit-cell is shown in Figure 7. It can be observed from Figure 7 that *S*_11_ and *S*_21_ parameters cross each other at 2 GHz and 4.8 GHz, respectively. This shows that the unit-cell structure has a bandgap between 2–4.8 GHz where electromagnetic waves are reflected, which makes the structure to have negative material properties [46,47].

**Figure 8:**
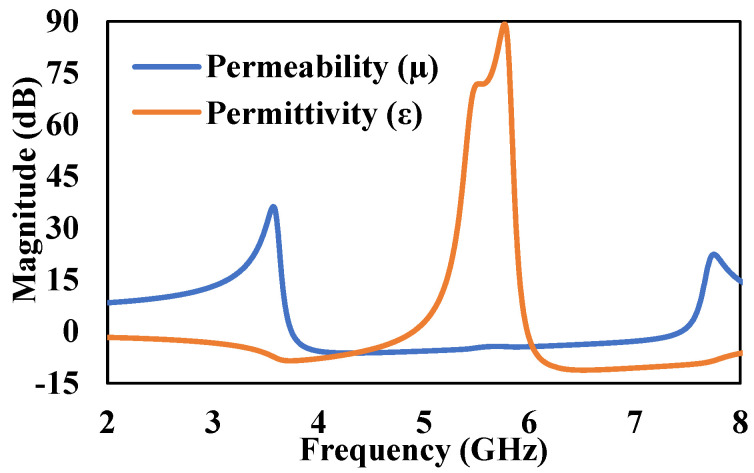
Simulated Permeability and Permittivity response of metallic-cross unit-cell.

Furthermore, effective permeability (*μ_eff_*) and permittivity (*ε_eff_*) of an equivalent MTM unit-cell are determined from Nicolson-Ross-Weir (NRW) approach [48]. The effective electromagnetic parameters (*μ_eff_* and *ε_eff_*) are generated from Scattering parameters, i.e., *S*_11_ and *S*_21_. The *μ_eff_* and *ε_eff_* has been calculated using the Equations (5)–(8) and its response with respect to frequency is shown in Figure 8. The values, *V*_1_ and *V*_2_ indicate the summation and difference of S-parameters. The *V*_1_ and *V*_2_ are calculated from Equations (5) and (6), respectively.

(5) $$V_{1}=S_{21}+S_{11}\;$$

(6) $$V_{2}=S_{21}-S_{11}$$

(7) $$\mu_{eff}=\frac{2}{jk_{0h}}\frac{1-V_{2}}{1+V_{2}}\;$$

(8) $$\varepsilon_{eff}=\frac{2}{jk_{0h}}\frac{1-V_{1}}{1+V_{1}}$$

where, *k*_0_ is wave number in free space and is equated to *ω*/*c*, *ω* is the radian frequency and *c =* 2*πf*, *c* is the speed of light *=* 3 × 10^8^ m/s, *μ_eff_* is the effective permeability of equivalent MTM, *ε_eff_* is the effective permittivity of equivalent MTM, and *h* is the thickness/height of the substrate.

### 2.3. Cross-Slot Metamaterial Structure Etched in the Ground Plane of Proposed Antenna

**Figure 9:**
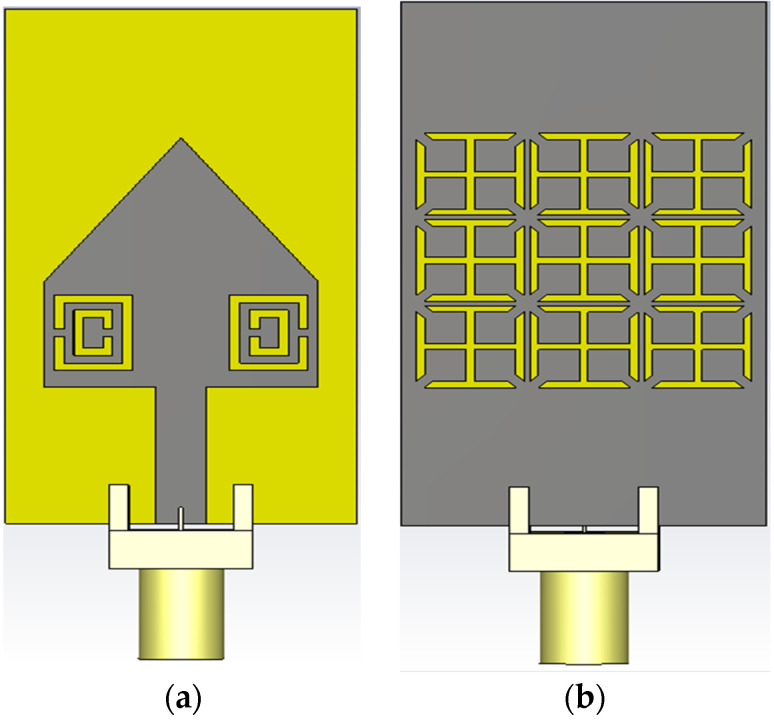
Layout of the proposed antenna with cross-slot MTM structure etched in the ground plane (**a**) Top Layer (**b**) Bottom layer.

**Figure 10:**
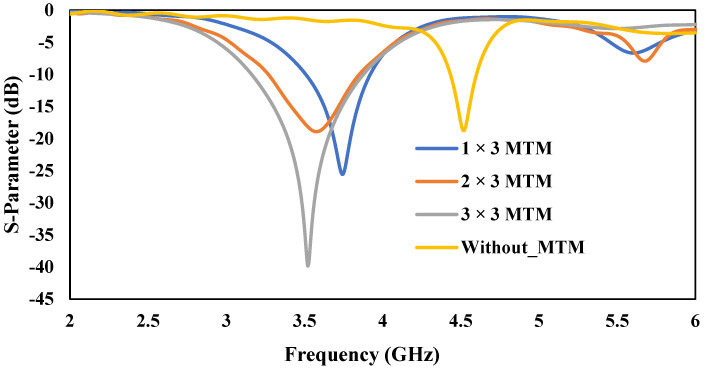
S-Parameter Response by varying the number of Cross-Slot MTM structure.

A 3 × 3 metallic-cross MTM structure has been etched in the ground plane of the proposed antenna to improve the bandwidth, as shown in Figure 9. After etching in the ground plane, this metallic-cross will be referred to as cross-slot MTM structure. The structure and dimension of the cross-slot MTM unit-cell are shown previously in Figure 5. A parametric analysis based on the number of cross-slot MTM structures has been investigated. The proposed antenna in Figure 9 without cross-slot MTM in the ground plane resonates at 4.5 GHz with a bandwidth of 150 MHz, as shown in Figure 10. Initially, a 1 × 3 cross-slot MTM structure was etched in the ground plane, which shows a bandwidth of 400 MHz. In order to further improve the bandwidth, a 2 × 3, and 3 × 3 MTM structure analysis was performed by etching in the ground plane. The final antenna design with 3 × 3 cross-slot MTM structure etched in the ground plane is shown in Figure 9b. The Cross-Slot unit-cell etched on the ground layer develops negative ε and *µ* [49]. The capacitance value of cross-slot unit-cell can be varied by changing the gap between them. By increasing the capacitance value, the bandwidth will improve [50]. So, by increasing the number of cross-slot unit-cell, the bandwidth of the antenna has been improved. From the S-parameter response shown in Figure 10, it was noticed that the bandwidth is gradually increasing as the number of MTM structure etching in the ground plane increases. Figure 10 shows the results of the 1 × 3, 2 × 3, and 3 × 3 MTM structure etched in the ground plane. It can be seen from the results that this MTM antenna design shows the bandwidth/return-loss characteristics of 400 MHz/−26 dB, 575 MHz/−20 dB and 730 MHz/−40 dB, respectively.

## 3. Analysis of Proposed Design with Different Substrate

**Figure 11:**
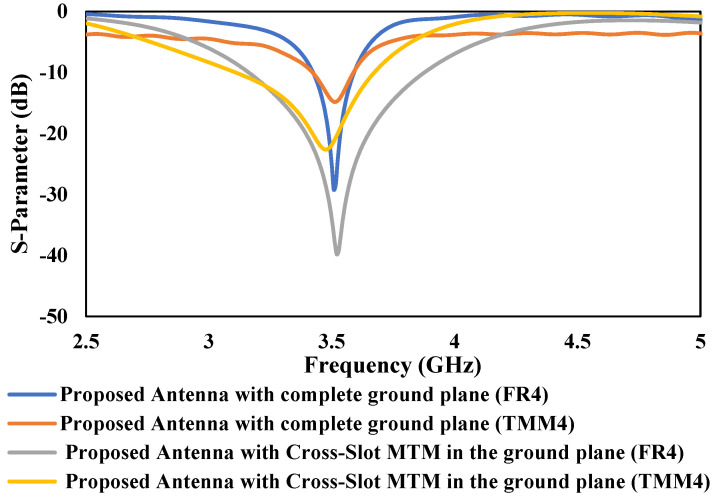
S-Parameter response of both proposed antennas using different substrate.

After getting the optimized dimensions of both proposed antennas from the simulation study, the return-loss (*S*_11_) plots of both MTM antennas are plotted using FR4 and Rogers TMM4 substrate, and the results are summarized in Figure 11. The *ε_r_* and *h* of the chosen Rogers TMM4 substrate were 4.5 and 1.524 mm, respectively. It is important to note that both substrates have very similar dielectric permittivity and thickness parameters. The purpose of selecting two different substrates, i.e., a high-end Rogers TMM4 and a low-end FR4 substrate, is to identify which one will provide more gain for the proposed antenna design.

**Figure 12:**
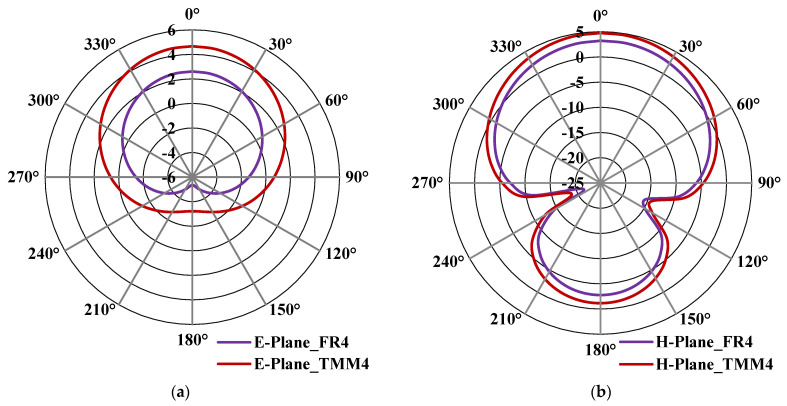
Simulated radiation pattern of the proposed antenna with a complete ground plane at 3.5 GHz (**a**) E-plane (**b**) H-plane.

**Figure 13:**
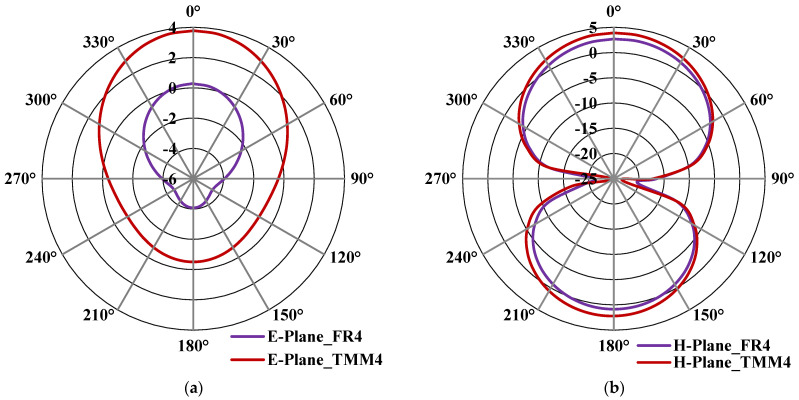
Simulated radiation pattern of the proposed antenna with cross-slot MTM structure etched in the ground plane at 3.5 GHz (**a**) E-plane (**b**) H-plane.

Figure 12a,b shows the E- & H-plane simulated radiation pattern results of the proposed antenna with the complete ground plane by using both FR4 and Rogers TMM4 substrates, which resonates at 3.5 GHz. From Figure 12a,b, it can be observed that the gain is improved from 2.9 dBi to 4.9 dBi by replacing the low-end and lossy FR4 substrate with the high-end Rogers TMM4 substrate. It is clear from this study that the proposed structure will provide high-gain characteristics by replacing the low-cost FR4 with high-end Rogers TMM4 substrate. Similarly, Figure 13a,b shows the E- & H-plane simulated radiation pattern results of the proposed antenna with cross-slot MTM structure etched on the ground plane using both substrates. This structure also exhibits better gain characteristics by replacing the substrate from FR4 with Rogers TMM4. The loss tangent (*tan δ*) of TMM4 and FR4 substrate are 0.002 and 0.025, respectively [51]. It means the FR4 substrate is a lossy substrate, and its radiation efficiency will always be less than the TMM4 substrate. This is the reason TMM4 substrate has a higher gain than FR4; even the dielectric permittivity and thickness parameters are the same. For the proof-of-concept in this work, the proposed antenna is fabricated using FR4 substrate due to its low-cost nature and easy availability.

## 4. Result and Discussion

**Figure 14:**
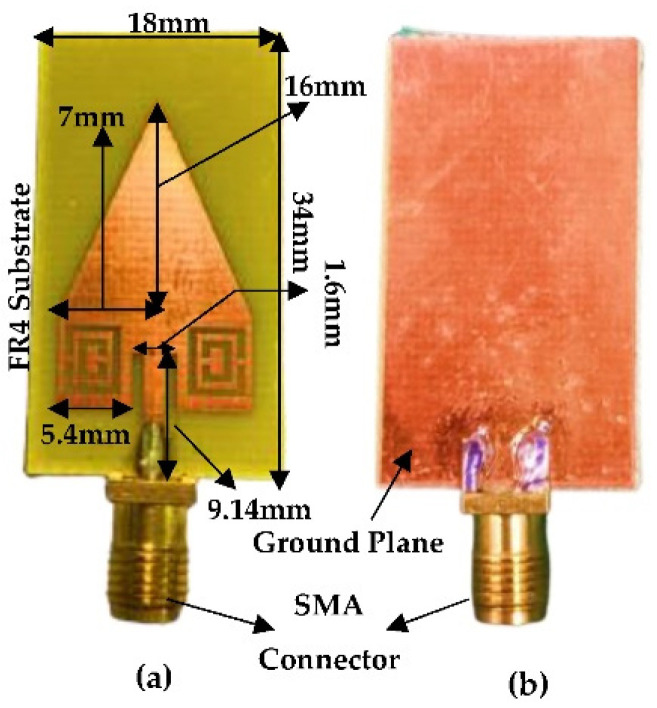
Fabricated structure of proposed antenna with complete ground plane (**a**) Top Layer (**b**) Bottom Layer.

**Figure 15:**
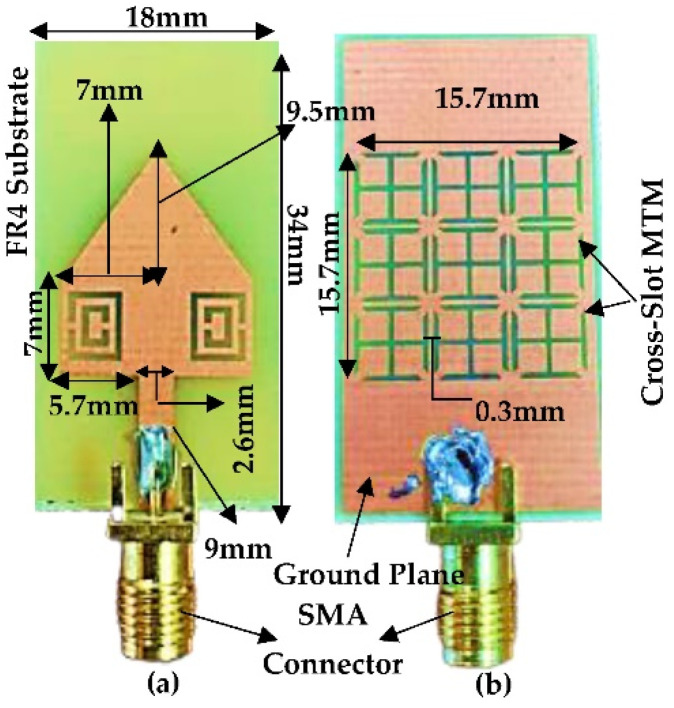
Fabricated structure of proposed antenna with cross-slot MTM structure etched on the ground plane (**a**) Top Layer (**b**) Bottom Layer.

The top layer in the proposed antenna design is based on the combination of RPA and TPA with CSRR structure, and the bottom layer has been modified by (1) complete ground plane, and (2) ground plane replaced by 3 × 3 cross-slot MTM structure. Figure 14a,b and Figure 15a,b show the fabricated antenna top and bottom layers of the proposed antenna with complete ground plane and proposed antenna with cross-slot MTM structure etched on the ground plane, respectively. The overall dimension of the antenna shows the compactness of the proposed design (18 × 34 mm^2^). Keysight (Agilent Technologies) FieldFox N9925A vector network analyzer (VNA) was used to conduct the S-parameter measurements of the fabricated antennas.

**Figure 16:**
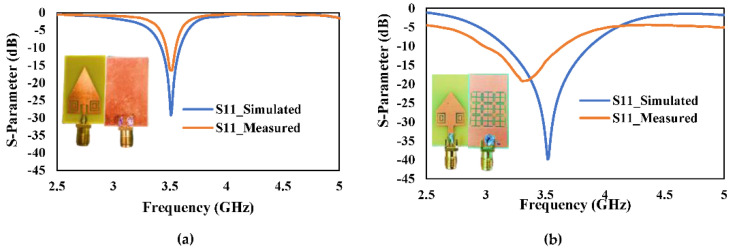
Simulated and Measured S-Parameter response (**a**) Proposed antenna with the complete ground plane (**b**) Proposed antenna with 3 × 3 cross-slot MTM structure etched on the ground plane.

Figure 16a,b shows the simulated and measured return-loss (*S*_11_) of the proposed antenna with (1) complete ground plane or (2) 3 × 3 cross-slot MTM etched on the ground plane. It can be observed from Figure 16a,b that both MTM antennas are resonating at 3.5 GHz and showing a measured return-loss of −16 dB and −19 dB, respectively. The MTM with complete ground plane and cross-slot MTM structure etched on the ground plane has a measured −10 dB bandwidth of 100 MHz (3.46 GHz–3.56 GHz) and 700 MHz (2.96 GHz–3.66 GHz), respectively.

**Figure 17:**
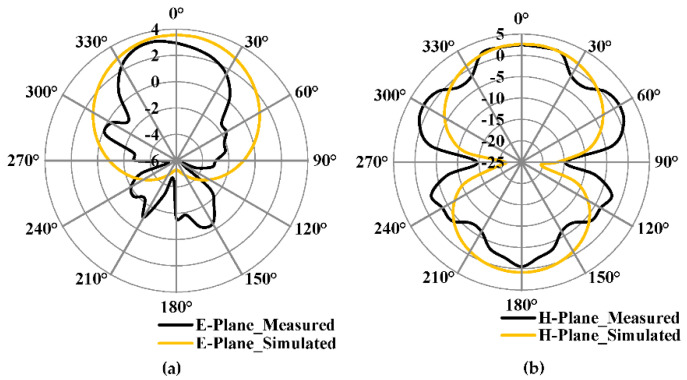
Simulated and Measured radiation pattern of the proposed antenna with complete ground plane (**a**) E-Plane (**b**) H-Plane.

The simulated and measured E- & H-plane radiation patterns proposed antenna with complete are shown in Figure 17a,b. The proposed antenna shows the maximum simulated and measured gain of 2.9 dBi and 2.6 dBi, respectively. From Figure 17a,b, it can be understood that the antenna radiates in the boresight. The measured radiation pattern slightly deviates from 0° as compared to the simulated result.

**Figure 18:**
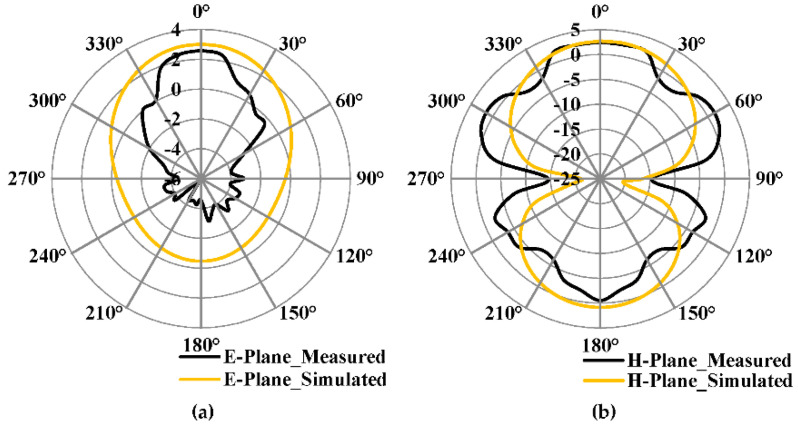
Simulated and Measured radiation pattern of the proposed antenna with 3 × 3 cross-slot MTM structure etched on the ground plane (**a**) E-Plane (**b**) H-Plane.

Figure 18a,b shows the E- and H-plane radiation pattern of the proposed antenna with 3 × 3 cross-slot MTM etched in the ground plane, respectively. It has a maximum simulated and measured gain of 2.5 dBi and 2.3 dBi, respectively, in the E-Plane. It has a side-lobe level of −2 dBi. The H-plane has a maximum simulated and measured gain of 2.45 dBi and 2.3 dBi, respectively. The gain of the fabricated prototype was measured using a standard horn antenna as a reference source.

**Figure 19:**
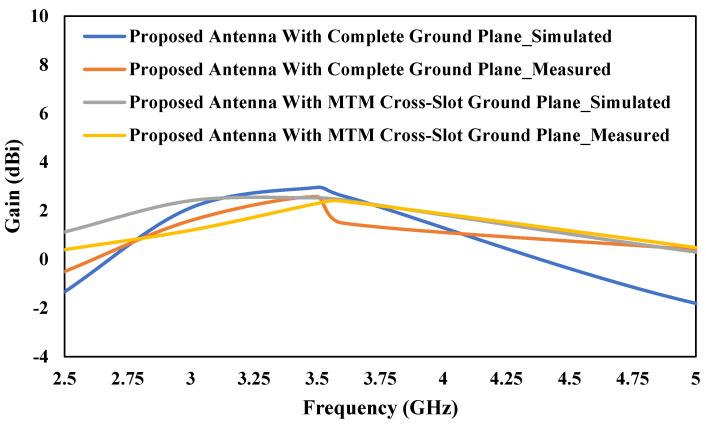
Simulated and Measured gain versus frequency graph for both proposed antennas.

Figure 19 shows the simulated and measured gain versus frequency graph for both proposed antennas. From the graph, it can be observed that the simulated and measured gain at 3.5 GHz is almost close to each other. The proposed antenna with complete ground plane has a little higher gain as compared to the antenna with a 3 × 3 cross-slot MTM structure. But the bandwidth is more for the antenna with 3 × 3 cross-slot MTM structure.

## 5. Comparison

Table 2 compares the proposed MTM antenna performance with several other MTM antennas presented in the literature and operating at 3.5 GHz [52,53,54,55,56,57,58]. All MTM antenna sizes and thicknesses in Table 2 are normalized to the free space wavelength *λ_o_* at the resonant frequency 3.5 GHz. The −10 dB relative bandwidth (BW) of these MTM antennas’ results are summarized in the BW column. Table 2 shows that previous MTM antenna designs either improved the bandwidth [52,53] or gain [54,55,56] or reduced the antenna size [56,57,58]. Although, the proposed MTM antenna based on cross-slot results shows a 60 percent reduction in size and a seven-fold increase in the bandwidth over the conventional MPA design at 3.5 GHz. The gain of the proposed antenna can be further improved by using a high-end Roger’s substrate. Many enticing features of this proposed MTM antenna include large bandwidth, high-gain, and compact size, making them an ideal candidate for the 5G IDAS.

## 6. Conclusions

A combination of triangular and rectangular patches with two kinds of MTM structure, which includes complementary split-ring resonator (CSRR) on the top layer and 3 × 3 cross-slot MTM on the bottom layer, was proposed in this study. The measured result of the FR4 substrate based proposed antenna with a cross-slot MTM structure etched on the ground plane shows larger bandwidth as compared to the MTM antenna with the complete ground plane. Both the antennas have a gain almost close to each other. Furthermore, when compared to the previous designs in Table 2, the MTM antenna with cross-slot etched on the ground plane exhibits larger bandwidth and better gain. The proposed MTM antenna with a cross-slot structure on the ground plane is considered the most promising candidate for 5G IDAS because of its miniaturized structure and is also considered a good choice for the upcoming 5G applications due to its small size, improved bandwidth, and gain. The gain can be boosted more in the future by adopting a high-end Rogers TMM4 substrate.

## Figures and Tables

**Table 1:** CSRR Unit-cell Dimensions.

CSRR Dimensions (Unit: mm)	
*l_c_*	5
*w_c_*	4
*g_c_*	0.5
*t_c_*	0.5
*s_c_*	0.5

**Table 2:** Performances comparison of existing MTM antenna with proposed structure at 3.5 GHz.

Ref./Year	Structure	Operating Freq. (GHz)	Measured Bandwidth (%)	Gain (dBi)	Antenna Size	Substrate Thickness	Relative Permittivity
[52]/2020	Two MTM SRR cells	3.5	28.4	1.9	0.34λ_o_ × 0.26λ_o_	0.018λ_o_	4.4
[53]/2017	CRLH Cells	3.5	14.1	1.6	0.40λ_o_ × 0.37λ_o_	-	4.4
[54]/2021	Triangular MTM patch antenna	3.5	2.1	6.3	0.58λ_o_ × 0.58λ_o_	0.017λ_o_	3
[55]/2021	Mushroom MTM	3.5	27.8	4.5	0.35λ_o_ × 0.35λ_o_	0.017λ_o_	2.2
[56]/2016	Complementary spiral resonators	3.5	1.43	5.72	0.16λ_o_ × 0.08λ_o_	0.024λ_o_	2.65
[57]/2021	Polygonal SRR	3.5	5.5	3	0.24λ_o_ × 0.35λ_o_	0.018λ_o_	4.4
[58]/2019	SRR-Loaded MTM	3.5	9.7	2.25	0.35λ_o_ × 0.36λ_o_	0.018λ_o_	4.6
This work/2022	CSRR-MTM	3.5	2.8	2.6	0.21λ_o_ × 0.39λ_o_	0.018λ_o_	4.3
This work/2022	CSRR-MTM with Cross-Slot MTM	3.5	20	2.3	0.21λ_o_ × 0.39λ_o_	0.018λ_o_	4.3
